# Supplementary material for: Multivariate analysis of disorder in metal–organic frameworks
Source: Nat Commun. 2022 Apr 21;13:2173. doi: 10.1038/s41467-022-29849-6 (PMC9023516; doi:10.1038/s41467-022-29849-6)
Supplement: Supplementary file 1 — Supplementary Information [file 41467_2022_29849_MOESM1_ESM.pdf]

Multivariate Analysis of Disorder in Metal–Organic Frameworks

SUPPLEMENTARY INFORMATION

Adam F. Sapnik,[1] Irene Bechis,[2] Alice M. Bumstead,[1] Timothy Johnson,[3] Philip A. Chater,[4] David A. Keen,[5] Kim E. Jelfs[2] and Thomas D. Bennett[1]*

1 Department of Materials Science and Metallurgy, University of Cambridge, Cambridge, CB3 0FS, United Kingdom.
2 Department of Chemistry, Imperial College London, Molecular Sciences Research Hub, London, W12 0BZ UK
3 Johnson Matthey Technology Centre, Blount's Court, Sonning Common, RG4 9NH, United Kingdom.
4 Diamond Light Source Ltd, Diamond House, Harwell Campus, Didcot, Oxfordshire, OX11 0DE, UK.
5 ISIS Neutron and Muon Facility, Rutherford Appleton Laboratory, Harwell Campus, Didcot, Oxfordshire, OX11 0QX, UK

* To whom correspondence should be addressed; E-mail: tdb35@cam.ac.uk

Supplementary Figures

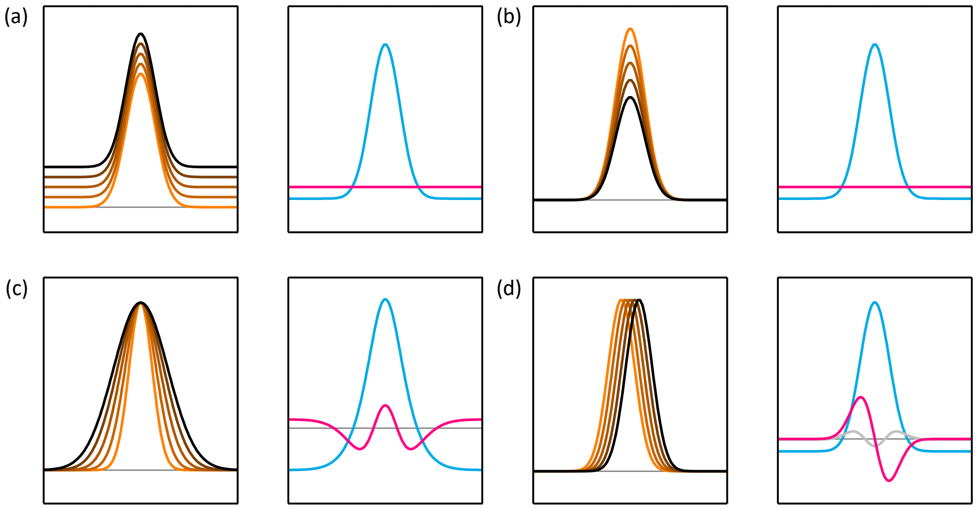

Supplementary Figure 1 Illustrative gaussian functions (orange to black) and their corresponding first (blue), second (pink) and third (grey) principal components. The Gaussian functions have been **(a)** vertically translated, **(b)** vertically scaled, **(c)** horizontally broadened, and **(d)** horizontally translated. In **(d)**, a third principal component is required due to the changing position of the peak, the contribution of which is not statistically significant for small translations.

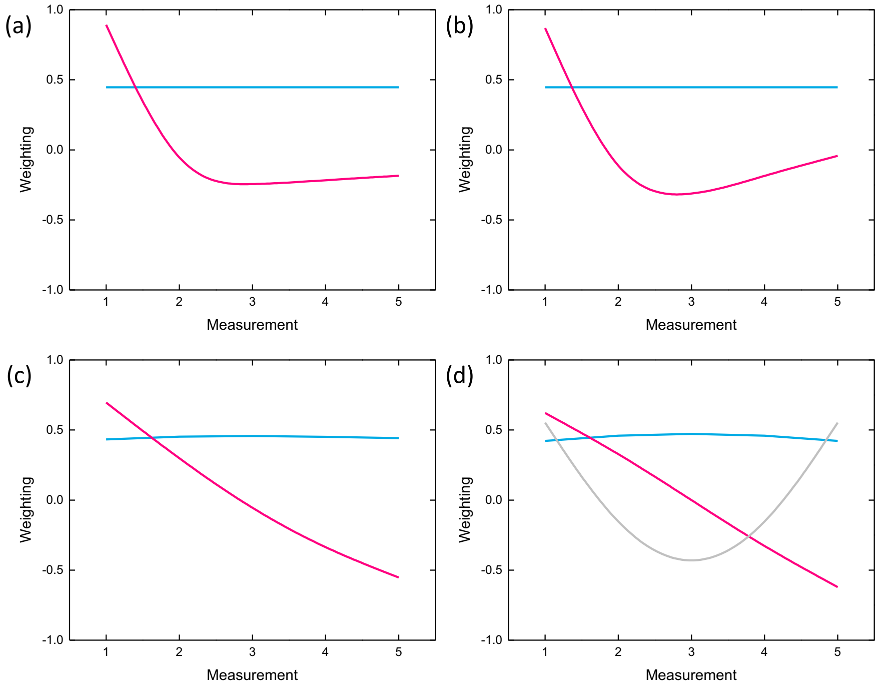

Supplementary Figure 2 Corresponding weightings of the primary (blue), secondary (pink) and tertiary (grey) components derived for the Gaussian functions in Supplementary Fig. 1 that have been **(a)** vertically translated, **(b)** vertically scaled, **(c)** horizontally broadened, and **(d)** horizontally translated. Measurement one corresponds to the original orange Gaussian function and five to the transformed black function.

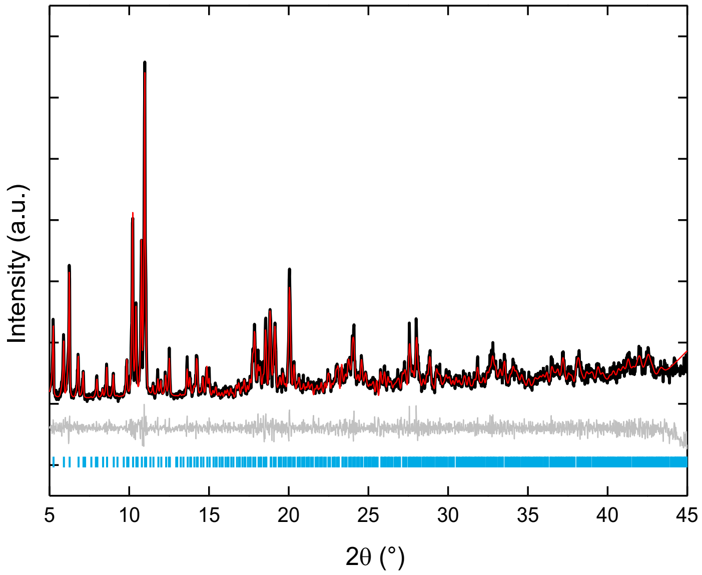

Supplementary Figure 3 Pawley refinement of MIL-100 (Fe). Experimental data (black), calculated diffraction pattern (red), difference function (grey) and symmetry-allowed reflections (blue). Symmetry allowed reflections were calculated from the reported crystallographic information file in Ref. 1.

Supplementary Table 1 Crystallographic data from Pawley refinement of MIL-100.

R_{wp} = 9.48	Experimental	Reported [1]
$a = b = c$	73.25(3)	73.340(1)
$\alpha = \beta = \gamma$	90	90

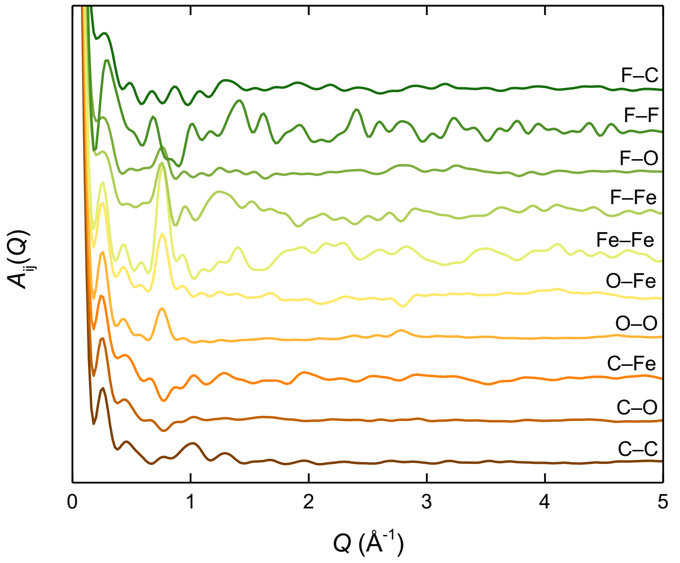

Supplementary Figure 4 Partial structure factors calculated from MIL-100 using RMCProfile.[2] The sharp increase at low-*Q* is due to additional low-angle scattering from the large crystallographic unit cell of MIL-100. H-containing partial structure factors have been omitted.

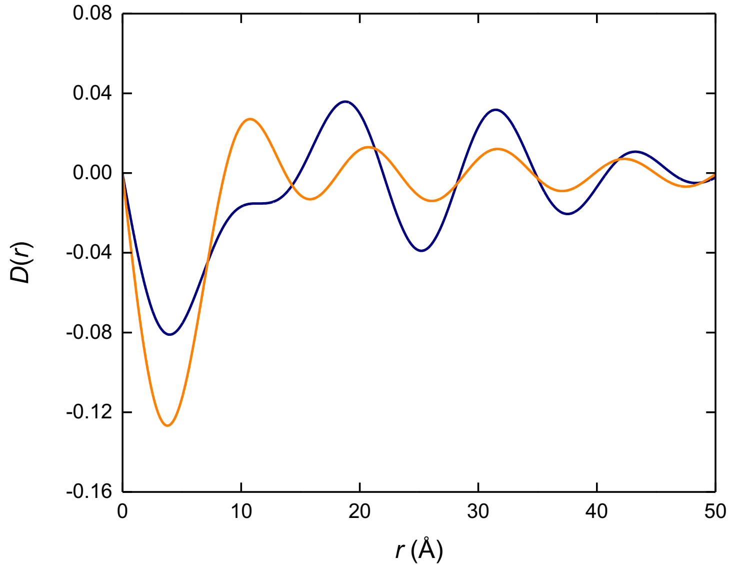

Supplementary Figure 5 Contribution of the lower-*Q* scattering to the PDF in MIL-100 (navy) and Fe-BTC (orange), obtained *via* the Fourier transform of the total scattering in the region 0.3 to 0.9 Å^{-1}.

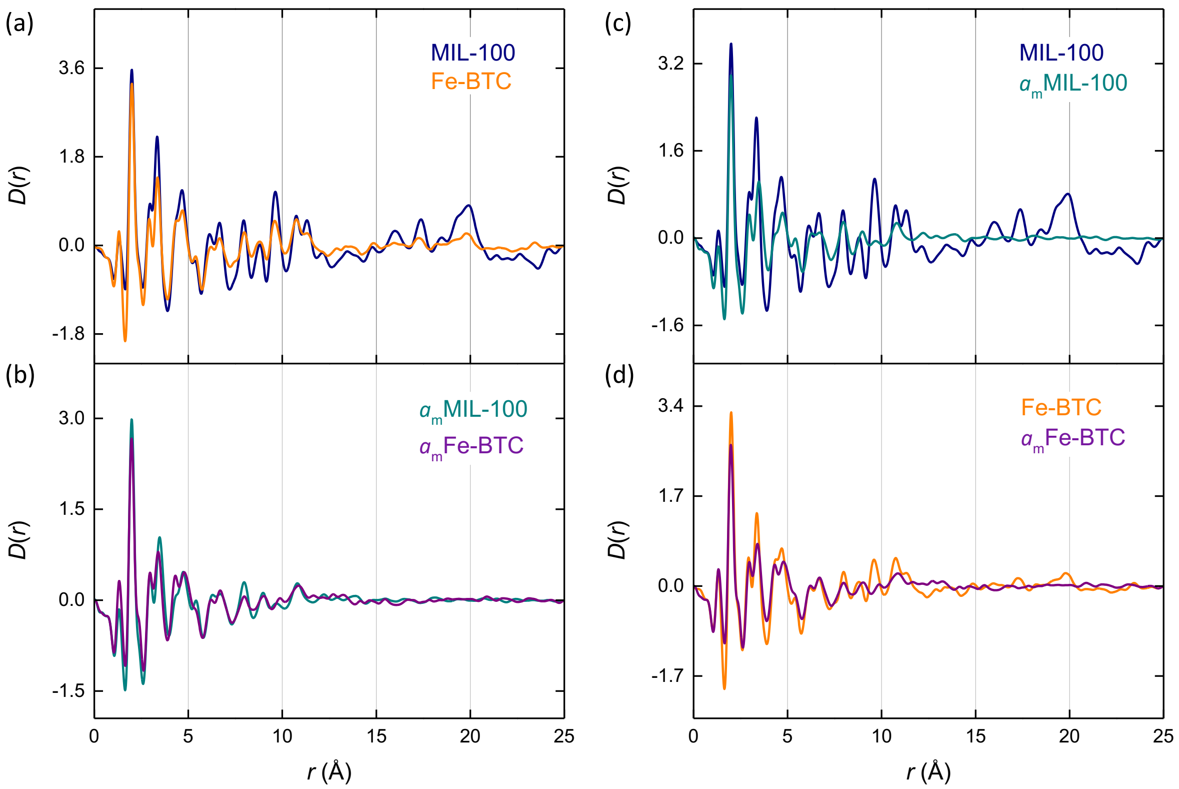

Supplementary Figure 6 Comparisons of the experimental PDF data: **(a)** the two starting materials, **(b)** the two materials obtained post-amorphisation, **(c)** MIL-100 pre- and post-amorphisation, and **(d)** Fe-BTC pre- and post-amorphisation.

Supplementary Table 2 Pearson's correlation coefficient for MIL-100 and Fe-BTC.

	MIL-100	Fe-BTC
**MIL-100**	1	0.770
**Fe-BTC**	0.770	1

Supplementary Table 3 Pearson's correlation coefficient for a_mMIL-100 and a_mFe-BTC.

	a_mMIL-100	a_mFe-BTC
**a_mMIL-100**	1	0.947
**a_mFe-BTC**	0.947	1

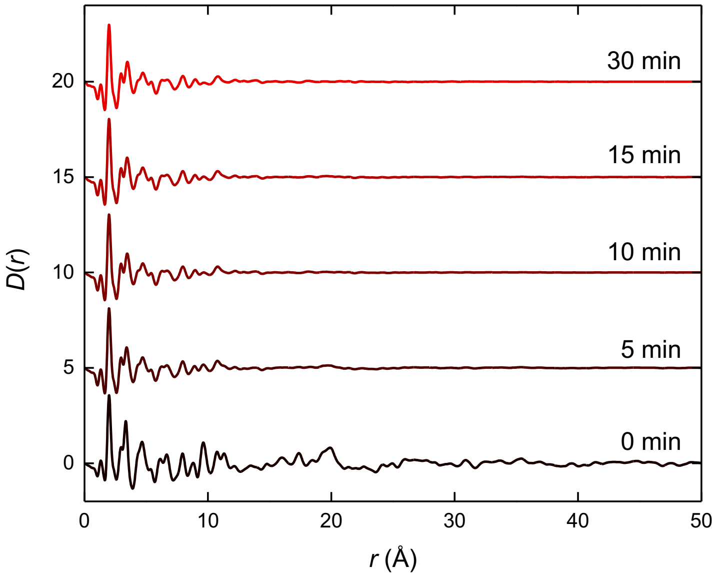

Supplementary Figure 7 *Ex situ* PDFs of the MIL-100 series, as in **Fig. 5a**, stacked for clarity.

Supplementary Table 4 Pearson's correlation coefficient for the series of MIL-100 materials.

MIL-100	0	5	10	15	30
**0**	1	0.834	0.741	0.756	0.715
**5**	0.834	1	0.988	0.990	0.979
**10**	0.741	0.988	1	0.999	0.999
**15**	0.756	0.990	0.999	1	0.998
**30**	0.715	0.979	0.999	0.998	1

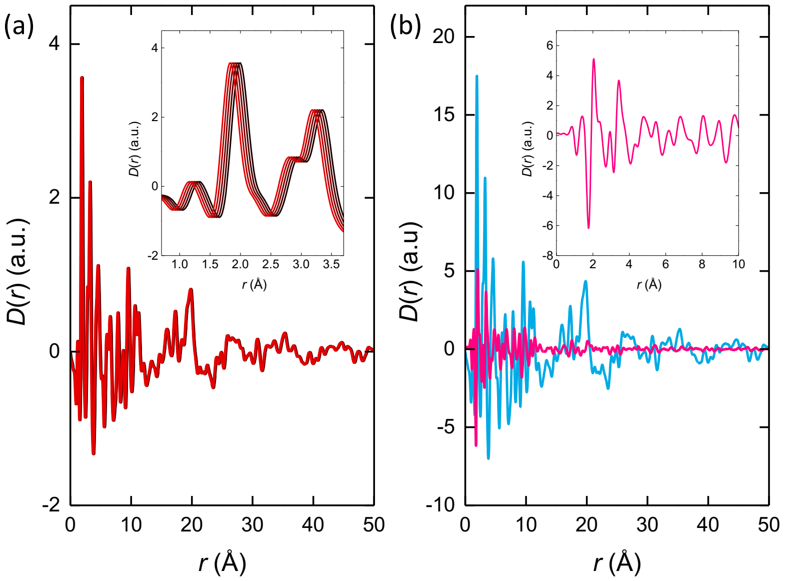

Supplementary Figure 8 (a) Synthetic dataset obtained *via* successive horizontal translation of the MIL-100 PDF. A representative total translation of 0.16 Å was used to replicate the observed shift in the experimental data at 3.32 Å. Inset shows the low-*r* region. **(b)** First (blue) and second (pink) principal components obtained *via* PCA of the synthetic data. Inset shows the low-*r* region of the second component.

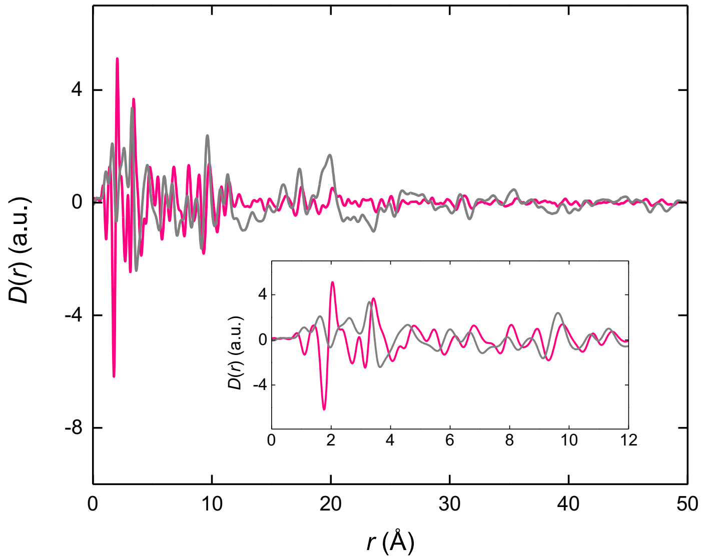

Supplementary Figure 9 Comparison between the second principal component obtained from the synthetic data (pink) in Supplementary Fig. 8 and the experimental collapse of MIL-100 (grey), as in the main text. Inset shows low-*r* region.

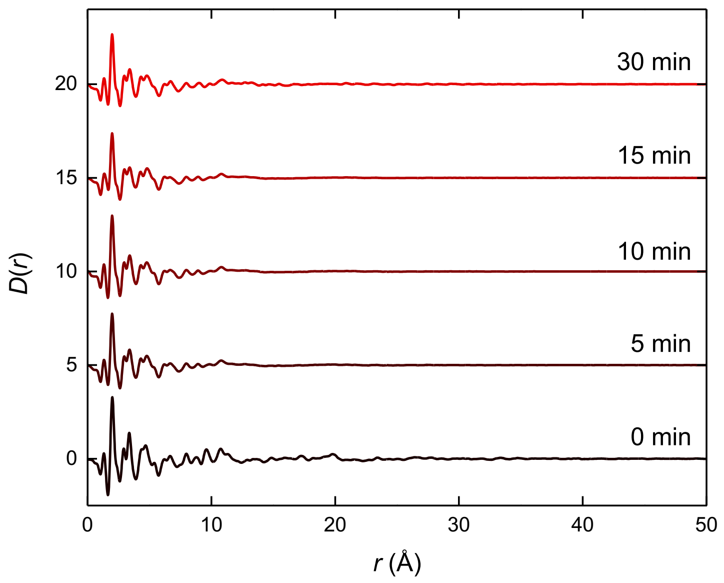

Supplementary Figure 10 *Ex situ* PDFs of the Fe-BTC series, as in Fig. 5d, stacked for clarity.

Supplementary Table 5 Pearson's correlation coefficient for the series of Fe-BTC materials.

Fe-BTC	0	5	10	15	30
**0**	1	0.939	0.939	0.889	0.929
**5**	0.939	1	0.995	0.986	0.996
**10**	0.939	0.995	1	0.971	0.993
**15**	0.889	0.986	0.971	1	0.981
**30**	0.929	0.996	0.993	0.981	1

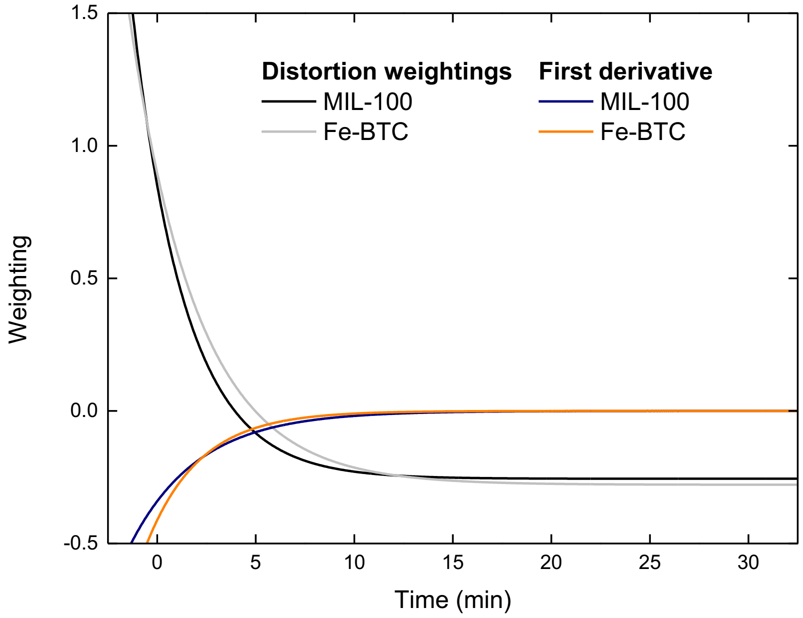

Supplementary Figure 11 Exponential decay curves fit to the weightings of the distortion component for MIL-100 (black) and Fe-BTC (grey). The first derivative of the exponential curves for MIL-100 (navy) and Fe-BTC (orange).

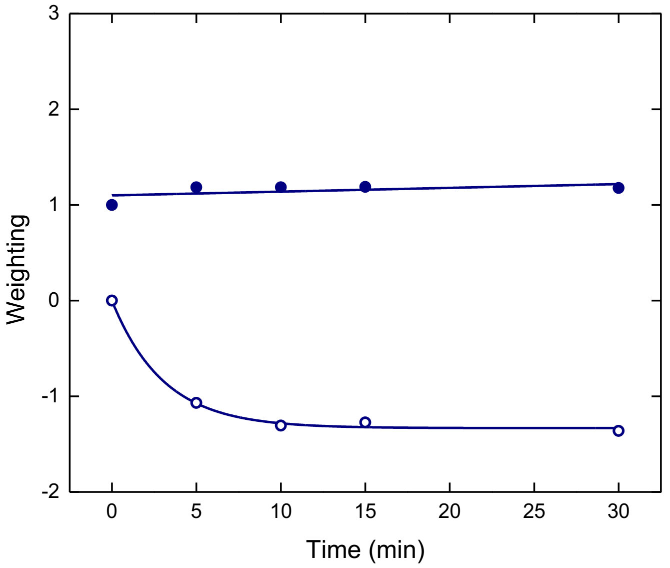

Supplementary Figure 12 Weightings obtained from the MIL-100 series when multiple linear regression is performed on the experimental data using MIL-100 and the second principal component (distortion).

Supplementary Table 6 Pearson's correlation coefficient for the series of TIF-4 materials.

TIF-4	25	100	200	300	400	420	440
**25**	1	0.993	0.986	0.940	0.844	0.843	0.840
**100**	0.993	1	0.983	0.945	0.858	0.857	0.855
**200**	0.986	0.983	1	0.973	0.882	0.881	0.881
**300**	0.940	0.945	0.973	1	0.955	0.955	0.954
**400**	0.844	0.858	0.882	0.955	1	0.999	0.999
**420**	0.843	0.857	0.881	0.955	0.999	1	0.999
**440**	0.840	0.855	0.881	0.954	0.999	0.999	1

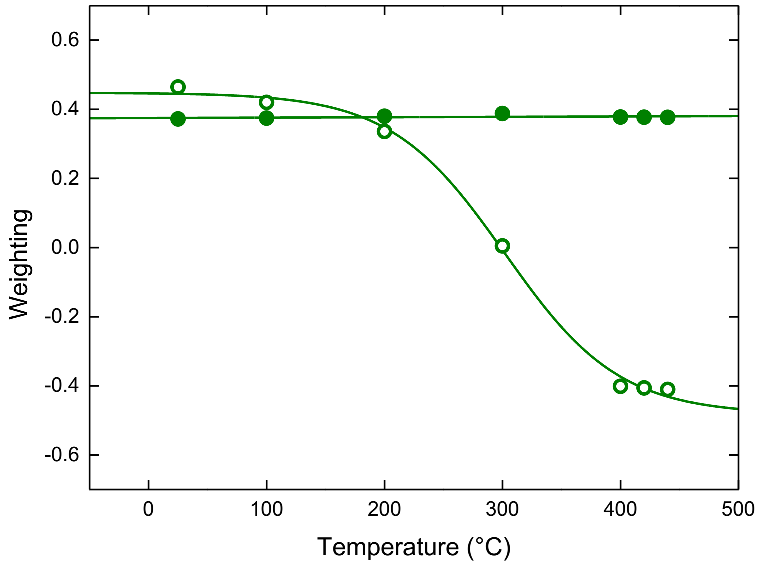

Supplementary Figure 13 Weightings obtained for the first (filled) and second (open) principal components in real space.

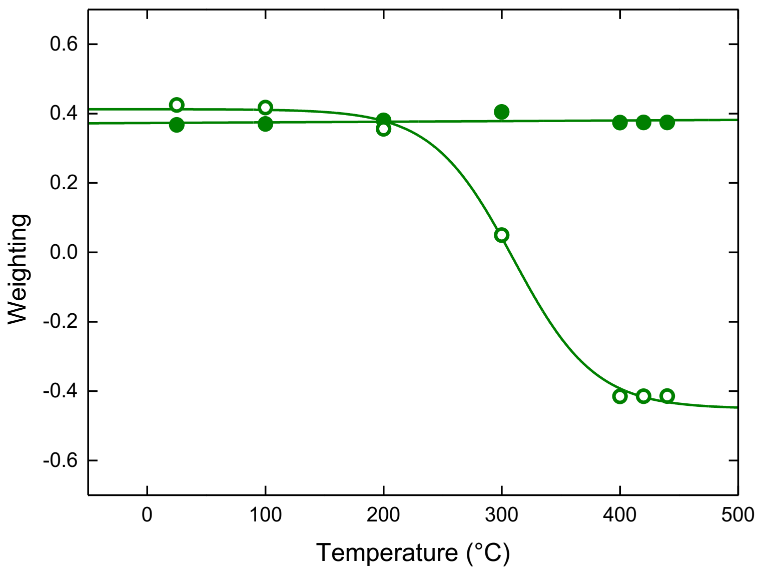

Supplementary Figure 14 Weightings obtained for the first (filled) and second (open) principal components in reciprocal space.

Supplementary References

1 Horcajada, P. *et al.* Synthesis and catalytic properties of MIL-100(Fe), an iron(III) carboxylate with large pores. *Chem. Commun.* **100**, 2820–2822 (2007).

2 Tucker, M. G. *et al.* RMCProfile: reverse Monte Carlo for polycrystalline materials. *J. Phys.: Condens. Matter.* **19**, 335218 (2007).
